# Atypical intraosseous mucoepidermoid carcinoma with two years of evolution and cutaneous infiltration

**DOI:** 10.4317/jced.55088

**Published:** 2018-11-01

**Authors:** Agnes Assao, Talita-da Silva-Nery de Souza, Diogo-Loureiro Freitas, Denise-Tostes Oliveira

**Affiliations:** 1 DDS, MSc, PhD Student, Department of Surgery, Stomatology, Pathology and Radiology, Area of Pathology, Bauru School of Dentistry, University of São Paulo, Bauru, São Paulo, Brazil; 2 DDS, Department of Surgery, Stomatology, Pathology and Radiology, Area of Pathology, Bauru School of Dentistry, University of São Paulo, Bauru, São Paulo, Brazil; 3 DDS, Private Practice, Cacoal, Rondônia, Brazil; 4 DDS, MSc, PhD, Department of Surgery, Stomatology, Pathology and Radiology, Area of Pathology, Bauru School of Dentistry, University of São Paulo, Bauru, São Paulo, Brazil

## Abstract

The intraosseous mucoepidermoid carcinoma is a rare lesion that frequently affects the posterior region of the mandible. This case reports a multilocular osteolytic radiolucency with two years of evolution, that expanded and perforated the cortical bone, with irregular and indefinite margins, that extended from the mandibular angle to the ascendant ramus, with cutaneous ulceration, detected in a 51 years-old male. An incisional biopsy was performed and confirmed the diagnosis of low-grade intraosseous mucoepidermoid carcinoma. The patient was submitted to partial mandibulectomy, neck dissection and post-operative radiotherapy. In three years of follow-up, there was no evidence of tumor recurrence. This case report reinforces that even a low-grade intraosseous mandibular mucoepidermoid carcinoma tends to expand and to perforate the bone cortical, infiltrating to adjacent soft tissues, in long time of evolution.

** Key words:**Intraosseous, mucoepidermoid carcinoma, mandible.

## Introduction

Mucoepidermoid carcinoma is the most common malignant salivary gland tumor that usually affects the major salivary glands, such as the parotid and submandibular glands, and less often, minor salivary glands (1). Clinically this tumor occurs as a painless swelling, frequently covered by normal oral mucosa (1,2). Histologically, most tumors are low-grade mucoepidermoid carcinomas and the prognosis is favorable for the patient (1).

The intraosseous mucoepidermoid carcinomas are rare tumors, usually of low-grade and less aggressive in nature (2). This tumor is more prevalent in the 3rd and 6th decades of life and females are more affected than males (2,3). As this tumor occurs in locals that do not normally contain salivary glands, its exact pathogenesis remains unknown (2-4). Imaginological findings include uni or multilocular radiolucent lesions that frequently affects the posterior region of mandible, including the mandibular angle (2-5). Clinically, expansion of the cortical bone, pain, paresthesia, trismus and dysphagia can be observed in intraosseous mucoepidermoid carcinoma (2,5,6).The prognosis of intraosseous mucoepidermoid carcinoma is favorable and cases with long term of evolution seem to present more aggressive clinical behavior (4,7,8).

The aim of this case report is to present an atypical intraosseous mucoepidermoid carcinoma in the posterior mandible with two years of evolution and cutaneous involvement. The clinical features, prognosis and treatment for advanced intraosseous mucoepidermoid carcinomas are the main subjects of discussion in this paper.

## Case Report

**Figure 1:**
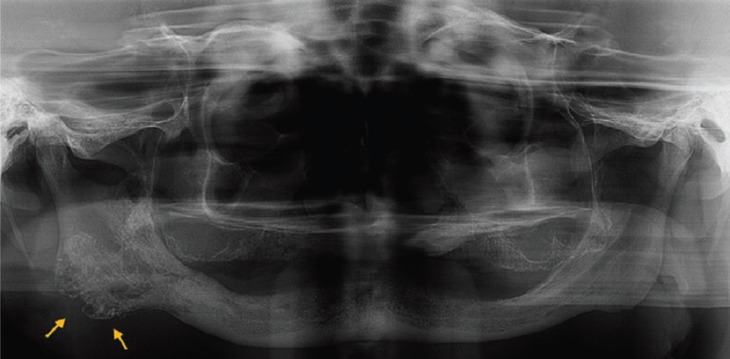
Panoramic radiography showing a multilocular osteolytic radiolucency at the right mandibular angle (arrows), with irregular and indefinite margins, extending to ascendant ramus.

**Figure 2:**
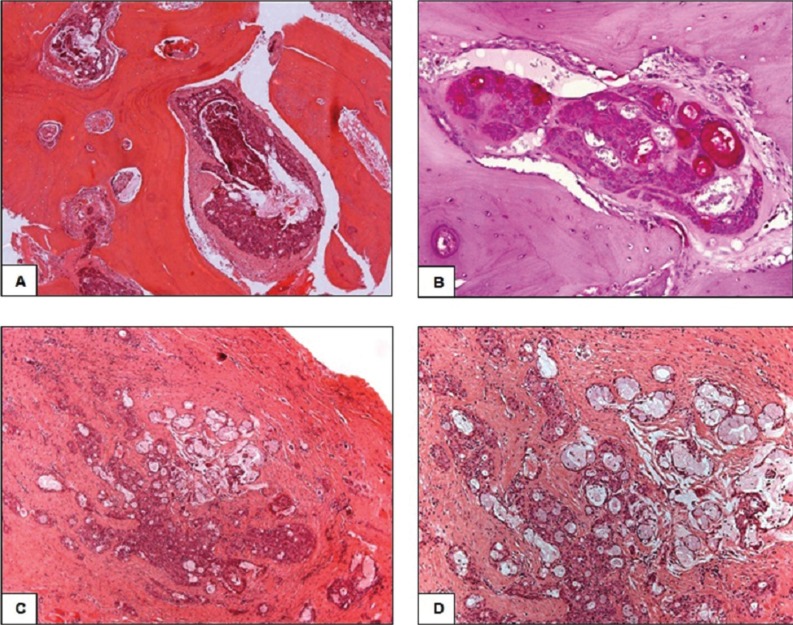
A: Histopathological finding showing epidermoid and mucous secreting neoplastic cells infiltrating the mandibular compact bone (Hematoxylin and eosin, Original magnification:200x). B: Details of mucous secreting neoplastic cells positive for periodic acid-Schiff staining (Periodic Acid-Schiff, Original magnification: 400x). C: Island of intermediate epithelial neoplastic cells with mucous and epidermoid appearance, infiltrating the soft tissue (Hematoxylin and eosin, Original magnification: 100x). D: Details of neoplastic epidermoid and mucous cells with moderate pleomorphism and discrete hypercromatism (Hematoxylin and eosin, Original magnification: 200x).

**Figure 3:**
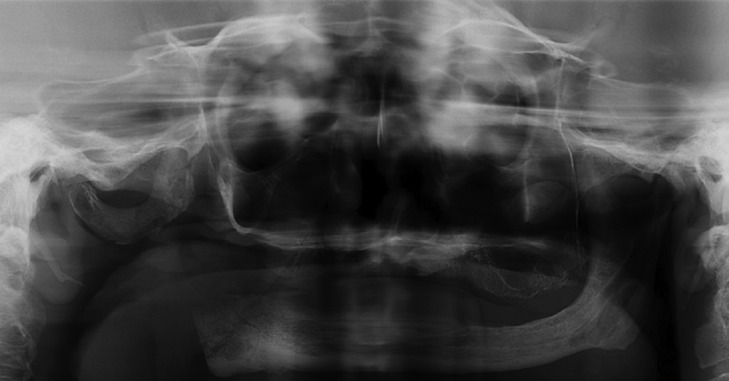
Post-operative panoramic radiography, with three-years of follow-up and no signs of tumor recurrence.

A 51-year-old male patient was referred to Hospital Municipal Rolim de Moura, Brazil, with chief complain of swelling and skin lesion in the region of right angle mandible, after a trauma during the feed, with two years of evolution. Consequently, severe trismus, limited mouth opening (approximately 10mm) and speaking difficulties were observed. The patient had the habit of smoking for 40 years and alcohol consumption for 30 years. Panoramic radiograph revealed edentulous maxilla and mandible, presenting a multilocular osteolytic radiolucency that expanded and perforated the bone cortical, with irregular and indefinite margins, extending from mandibular angle into ascendant ramus. The mandibular angle was almost entirely occupied by lesion (Fig. 1). Extra-oral examination revealed swelling in mandible right region and an ulcerated area in cutaneous region. Fine needle aspiration was negative and an incisional biopsy was performed. Clinical hypothesis was of osteosarcoma or benign odontogenic tumor. Microscopic analysis showed cystic formations and nests of intermediate epithelial neoplastic cells with mucous and epidermoid appearance, inside the viable compact bone extending into surrounding soft tissue. Some of the malignant cells presented moderate pleomorphism and discrete hypercromatism. The cystic spaces were lined by mucous secreting cells positive for periodic acid-Schiff staining (Fig. 2). Based on clinical, radiographic and microscopic features, the final diagnosis was of intraosseous low grade mucoepidermoid carcinoma. The patient was submitted to surgical partial mandibulectomy including the removal of part of the tongue, floor of the mouth and ipsilateral neck dissection of the lymph node. The histopathological analysis of the surgical specimen confirmed intraosseous low grade mucoepidermoid carcinoma, with surgical margins and lymph nodes free of the tumor. Six months after tumor resection, the patient was submitted to another surgery with rotation of pectoral flap, due to bone exposure. Adjuvant radiotherapy was prescribed for sixty days. Three-years follow-up showed no sign of tumor recurrence and the patient is being accompanied for rehabilitation (Fig. 3).

## Discussion

Intraosseous mucoepidermoid carcinomas, such as the case reported, rarely occurs in the jaws and the mandible is more affected than maxilla (2,5,9). According to the well-established clinical and demographic features of mandibular intraosseous mucoepidermoid carcinomas, described in English literature ( Table 1), from eight selected studies (2,3,5-7,9-11), including two retrospective studies, the tumor presents higher prevalence in males with age varying from 14 to 78 years-old (2,6,9,10). As in our case reported, the body and the posterior region of mandible are more commonly affected by intraosseous mucoepidermoid carcinomas, as described in Table 1,and only one patient developed the tumor in anterior region of the mandible (9).

**Table 1:**
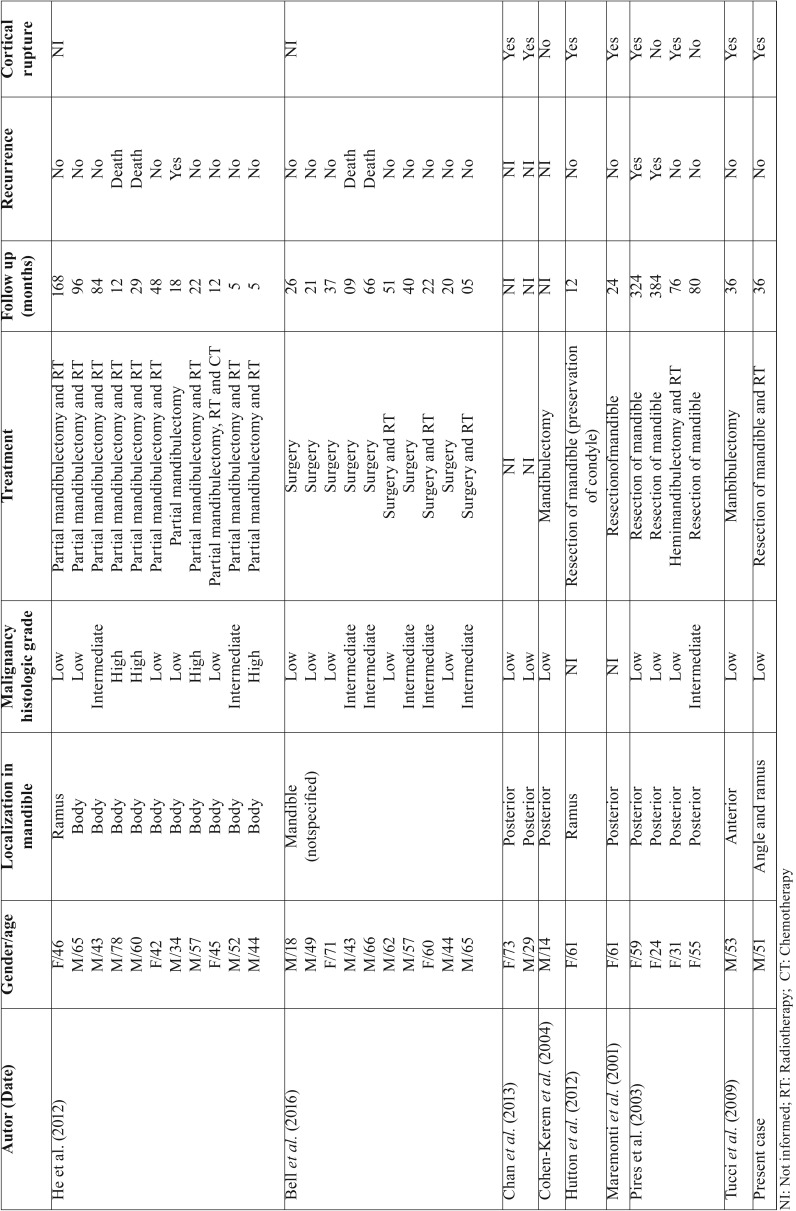
Clinical and pathological features of 32 reported cases of mandibular intraosseous mucoepidermoid carcinomas.

The clinical behavior and the prognosis of the intraosseous mucoepidermoid carcinomas are variable. Although one of the accepted criteria (12) to classify this tumor is the integrity of osseous cortical margins, other authors (7,9)already confirmed that the absence of rupture of cortical plate is not essential for diagnosis of intraosseous mucoepidermoid carcinomas. In addition, it has been suggested a positive correlation between radiographic findings of irregular margins, cortical destruction and invasion of intraosseous mucoepidermoid carcinomas with more aggressive grade tumor and unfavorable prognosis (7).

Curiously, in our literature review ( Table 1), including the present case reported, from nine studies of intraosseous mucoepidermoid carcinomas with rupture of mandibular cortical, most of them were classified as tumor of low-grade of malignancy (3,5,7,9-11). Based on the clinical features described above, including our case of low-grade intraosseous mucoepidermoid carcinoma with two years of evolution, we can suppose that the progression of this tumor over time tends to cause expansion and perforation of bone cortical, extending to soft tissue adjacent such as oral mucosa and skin (3,5,7,9).

Concerning management and prognosis of the 32 cases of mandibular intraosseous mucoepidermoid carcinomas, three of them classified as low-grade recurred, as described in Table 1. Although these cases had been surgically treated and followed for long time (up to 18 months), the complementary post-operative radiotherapy was not performed, which probably influenced the patients’ prognosis.

In addition, taking into account, in our case reported, the aggressive clinical signs of tumor evolution, as size of the lesion, proximity to important structures (mandible condyle), cortical perforation, cutaneous ulceration and clinical lymph node involvement, the treatment of the patient with mandibular intraosseous mucoepidermoid carcinoma consisted of partial mandibulectomy and neck dissection associated with post-operative radiotherapy for sixty days. The three-years of follow-up showed no sign of tumor local or regional recurrences.

According to He *et al.* (2012), the surgical resection of the tumor, including total maxillectomy for large tumors (>2x2cm) and partial maxillectomy for small tumors (<2x2cm) with negative margins accompanied by post-operative radiotherapy is recommended in order to improve prognosis(6). The authors found a better survival rate (72.7%) in those patients that received radiotherapy.

Microscopically, it is important to distinguish the low-grade intraosseous mucoepidermoid carcinomas from glandular odontogenic cysts. The glandular odontogenic cysts are characterized by the presence of a thin cystic lining with cuboidal cells and papillary intraluminal proliferation (7). On the other side, the presence of epidermoid, mucous and clear cells, permeated by cystic spaces and entrapped by solid viable bone, characterizes the intraosseous mucoepidermoid carcinomas (Fig. 2). Additionally, the solid epithelial proliferation is the main distinguishing feature, not seen in glandular odontogenic cysts and imperative for intraosseous mucoepidermoid carcinoma diagnosis (7).

Concluding, the present case reported reinforces that although low-grade intraosseous mucoepidermoid carcinomas have a good prognosis, a careful evaluation and the overlapping clinical and microscopic features of each case is necessary. Furthermore, the early detection of the intraosseous mucoepidermoid carcinoma is important to reduce the morbity and to improve the patient’s prognosis.
